# Essential Oils and Bioactive Components against Arthritis: A Novel Perspective on Their Therapeutic Potential

**DOI:** 10.3390/plants9101252

**Published:** 2020-09-23

**Authors:** Mariangela Marrelli, Valentina Amodeo, Maria Rosaria Perri, Filomena Conforti, Giancarlo Statti

**Affiliations:** Department of Pharmacy, Health and Nutritional Sciences, University of Calabria, 87036 Rende (CS), Italy; valentina.amodeo@unical.it (V.A.); mariarosaria.perri@unical.it (M.R.P.); filomena.conforti@unical.it (F.C.); giancarlo.statti@unical.it (G.S.)

**Keywords:** anti-arthritic, anti-inflammatory, essential oils, medicinal plants, rheumatoid arthritis, secondary metabolites, terpenes

## Abstract

Essential oils (EOs) are known to possess a number of beneficial properties. Their antimicrobial, anti-inflammatory, antioxidant, antidiabetic, and cancer-preventing activities have been extensively reported. Due to their wide use as food preservers and additives, as well as their use in agriculture, perfumes, and make-up products, these complex mixtures of volatile compounds have gained importance from a commercial point of view, not only in the pharmaceutical industry, but also in agronomic, food, cosmetic, and perfume industries. An analysis of the recent scientific literature allowed us to highlight the presence of an increasing number of studies on the potential antiarthritic properties of EOs and their main constituents, which seems to suggest a new interesting potential therapeutic application. The aim of this review is to examine the current knowledge on the beneficial effects of essential oils in the treatment of arthritic diseases, providing an overview of the reports on the in vivo and in vitro effects of EOs. Furthermore, this review critically examines the recent findings on the potential roles of the main components of EOs in the exerted beneficial effects. Obtained negative results are also reported.

## 1. Introduction

Arthritis is one of the most common chronic health problems and a major cause of disability [1]. The term “arthritis” derives from the Greek words “arthron” and “ites”, which mean inflammation of the joint. It can be defined as a chronic, inflammatory, and systemic autoimmune disorder characterized by pain, swelling, and rigidity of the synovial joints [2].

Arthritis includes more than 100 different disorders, with osteoarthritis (OA), rheumatoid arthritis (RA), psoriatic arthritis, gout, and fibromyalgia being the most common types [3].

Osteoarthritis is the most frequent form of arthritis [4]. This degenerative disease is characterized by damage to the articular cartilage of the knee, hip, and others lower extremity joints. The estimated risk for knee OA is approximately 47% in women and 40% in men, and its prevalence is projected to increase because of the ageing of the population and the growing occurrence of obesity [5]. Rheumatoid arthritis is a chronic and systemic inflammatory autoimmune disorder in which joint inflammation leads to cartilage and bone damage, disability, and even to systemic complications and enhanced morbidity and mortality [6]. A number of factors, such as genetic factors, the environment, and autoimmunity, play roles in RA pathogenesis [7]. All these factors induce the activation of the immune system, which cause autoantigen presentation with antigen-specific T and B cells activation and aberrant inflammatory cytokines production, thus leading to synovitis and cartilage and subchondral bone destruction. Extra-articular organs, such as the skin and the cardiovascular system, can also be involved [6].

The pharmacological management of both these major forms of arthritis is mostly symptomatic and is aimed at reducing pain and inflammation. This includes the use of non-steroidal anti-inflammatory drugs (NSAIDs) and oral glucocorticoids. Current treatment modalities for RA are based on the use of disease-modifying antirheumatic drugs (DMARDs), such as methotrexate, hydroxychloroquine, and sulfasalazine. However, these drugs can cause serious side effects, including gastrointestinal complaints, pneumonitis, and liver function abnormalities. More recently, biologic drugs (antibodies or soluble receptors for IL-1, IL-6, and TNF-a) have gained importance in the treatment of OA and RA. These agents are able to reduce inflammation and joint destruction, however besides the higher cost of such new agents, which limits their use, both their long-term side effects and benefits have not yet been fully understood. These drugs are not always effective and severe side effects may occur, such as serious infections and increased risk of cancer. Immunosuppressive and cytotoxic drugs (e.g., leflunomide, azathioprine, cyclosporine, and cyclophosphamide) may be used as well, but these drugs also cause a number of toxic side effects [8,9].

As a consequence, the use of botanicals and nutritional supplements has gained importance, and the number of studies on the potential health benefits of plant extracts in the treatment of arthritis is increasing [10].

The antiarthritic potential of plant species and extracts has been extensively reviewed in the last decade, and a number of papers dealing with such botanicals have been published [11,12,13,14,15,16,17,18,19,20].

In contrast, the present review aims to specifically offer an overview of the plant essential oils (EOs) with antiarthritic potential.

Essential oils are complex mixtures containing low molecular weight compounds, mainly monoterpenes, sesquiterpenes, and oxygenated derivatives, extracted from aromatic plants [21]. A number of biological properties have been detected for EOs, such as antimicrobial, antioxidant, cancer-preventing, antimutagenic, antidiabetic, and anti-inflammatory properties [22,23,24].

The analysis of the most recent literature allowed us to identify an increasing number of studies focusing on the in vitro and in vivo antiarthritic properties of essential oils. Overall, these findings give interesting perspectives on the therapeutic use of EOs and their chemical constituents.

## 2. Methods

This search, conducted over a period of 5 months, included references published up until July 2020. The academic search engines Google Scholar, Pubmed, Microsoft Academic, and Scopus were utilized. The keywords “essential oils”, “arthritis”, “antiarthritic”, “anti-rheumatoid arthritic effect”, “terpenes”, and “volatile oil” were searched in the databases. These descriptors were combined by using Boolean operators, e.g., “and”, “+”, allowing for more targeted results. Studies focusing on the antiarthritic potential of essential oils in their whole forms or their single pure active components were included in the research, and both in vitro and in vivo studies were taken into account.

## 3. Essential Oils with Antiarthritic Potential

**Figure 1:**
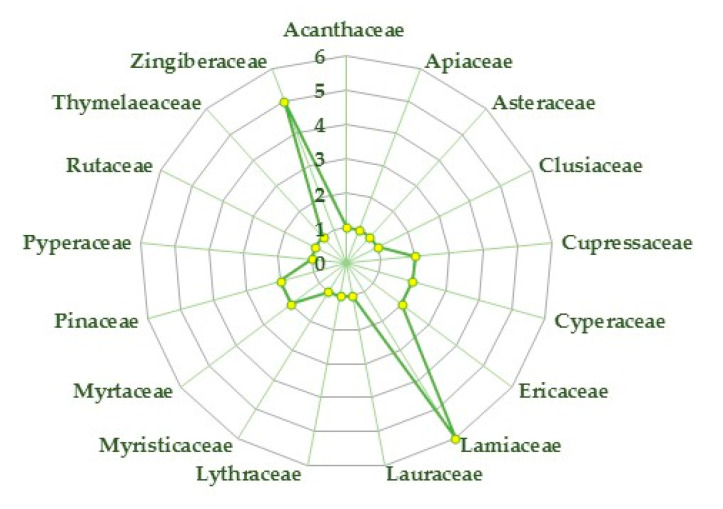
Number of plant species whose essential oils (EOs) showed antiarthritic potential for the different families.

The purpose of this review is to provide an overview of the studies dealing with the potential beneficial effects of EOs and their phytochemical constituents on arthritic conditions. A total of 31 EOs from different plant species have been reported in the literature to have in vitro or in vivo effectiveness. The investigated EOs that have been proved to have antiarthritic potential belong to a number of plant families (Figure 1), mainly Lamiaceae (6 species) and Zingiberaceae (5), followed by Cupressaceae, Cyperaceae, Ericaceae, Myrtaceae, and Pinaceae (2).

### 3.1. Aquilaria agallocha *Roxb.*

*Aquilaria agallocha* Roxb. (Thymelaeaceae), commonly known as “agarwood”, grows wild in the Himalayan region and East India, and it is cultivated in India, Bangladesh, Indonesia, and Sri Lanka [25]. Agarwood EOs and extracts contain a complex mixture of sesquiterpenes and chromones. Depending on the extraction technique, fatty acids and phenols have also been identified. A number of biological properties have been reported for this species, such as antioxidant, antimicrobial, antidiabetic, and antinociceptive properties [26,27].

Rahman and colleagues reported the anti-arthtritic potential of *A. agallocha* Roxb. heartwood essential oil (EO) [28]. In vitro, at the concentration of 500 µg/mL, this essential oil was demonstrated to induce 56.71% inhibition of protein denaturation. The biological potential was also assessed in vivo, in Freund’s adjuvant-induced arthritic rat model. At doses of 125 mg/kg and 250 mg/kg, *A. agallocha* EO inhibited the increase in paw volume, with maximum inhibition values equal to 19.78% and 27.88%, respectively.

### 3.2. Cedrus deodara *(Roxb.) Loud.*

*Cedrus deodara* (Roxb.) Loud. (Pinaceae), commonly named deodar, is a large evergreen tree native to the western Himalayas, traditionally used in Ayurvedic medicine for the treatment of inflammatory diseases, such as arthritis and bronchitis [29,30].

Shinde and coworkers investigated the anti-inflammatory activity of *C. deodara* wood EO in adjuvant arthritic male albino rats [31]. The oil (50 and 100 mg/kg) was administered orally 5 days prior to injection of FCA, and it was administered until day 30. *C. deodara* wood EO inhibited the acute phase of adjuvant-induced response (first 7 days following the injection of FCA). Moreover, the EO showed analgesic activity against acetic-acid-induced writhing and hot plate reaction in mice.

### 3.3. Chamaecyparis obtusa *(Siebold & Zucc.) Endl.*

Suh and colleagues tested the effects of EO from the leaves of *Chamaecyparis obtusa* (Siebold and Zucc.) Endl. (Cupressaceae) on pain-related behavior and pro-inflammatory cytokines in rats with carrageenan-induced arthritis [32]. The obtained results demonstrated the anti-inflammatory and antinociceptive effects of this essential oil. The intra-articular application of *C. obtusa* EO inhibited pain-related behavior and the expression of pro-inflammatory cytokines TNF-α, IL-1β, IL-6, and COX-2 in inflamed knee joints in rats.

### 3.4. Cyperus *spp.*

Biradar and coworkers investigated the in vivo antiarthritic potential of the EOs from two *Cyperus* species (Cyperaceae) from India, *C. esculenthus* L. and *C. rotondus* L. [33]. Samples were tested using the formaldehyde-induced arthritis model in Wistar albino rats. EOs were administered at the dose of 500 mg/Kg, and after 10 days of treatment they were able to inhibit paw edema by 75.54% (*C. rotondus*) and 76.58% (*C. esculenthus*).

### 3.5. Gaultheria fragrantissima *Wall.*

*Gaultheria fragrantissima* Wall. is an evergreen shrub belonging to the Ericaceae family that is widely distributed in the Himalayan region and northeastern India. Its EO has been reported to have antioxidant, antibacterial, insecticidal, and nematicidal activities [34].

Uriah and colleagues evaluated the in vitro biological potential of the EO obtained from the leaves of *G. fragrantissima* [35]. The antiarthritic activity was evaluated with the egg albumin denaturation method and the protein denaturation method using bovine serum albumin as a protein model. At a concentration of 250 µg/mL, the sample induced 49.86% inhibition of protein denaturation.

### 3.6. Lagerstroemia speciosa *(L.) Pers.*

*Lagerstroemia speciosa* (L.) Pers. is a common ornamental tree belonging to the Lythraceae family. The leaves are known to contain triterpenes, tannins, and flavones [36]. *L. speciosa* has been studied for its beneficial effects towards glucose and lipid metabolism [37]. The in vitro antiarthritic potential of the EOs obtained from fresh and dried leaves of this species was investigated by means of the protein denaturation method, using abumin as a protein model [38]. Samples were tested at concentrations ranging from 50 to 800 μg/mL, which were both able to inhibit protein denaturation.

### 3.7. Litsea cubeba *(Lour.) Pers.*

*Litsea cubeba* (Lour.) Pers. (Lauraceae) is a shrub or tree distributed in southeastern Asia. It is well known for the EO that can be extracted from the different parts of the plant, with various compositions and yields. The fruit EO is a major source for citral, which is widely used as an aroma additive in food and cosmetics [39].

The anti-arthritis potential of *L. cubeba* EO was tested by Zhao and coworkers [40]. Limonene, α-citral, and β-citral were the main components in the investigated essential oil. The antiarthritic potential was verified in vivo in collagen-induced arthritic rats. The treatment with EO caused the reduction of paw swelling and serum inflammatory factor levels.

### 3.8. Ocimum americanum *L.*

*Ocimum americanum* L. (Lamiaceae) is an annual plant native to Africa, also named American basil, which is commonly used for its flavor properties [41,42]. The antiarthritic potential of *O. americanum* L. fresh leaves EO was tested in vivo in mice in an experimental model of zymosan-induced arthritis [43]. The EO was characterized by the presence of linalool, eugenol, 1,8-cineole, and camphor as the major compounds. At doses of 150 and 300 mg/kg, *O. americanum* EO significantly inhibited leukocyte migration to the articular cavity in the knee joint of zymosan-induced arthritic Balb/c mice. Moreover, at 150 mg/kg the EO was able to reduce paw edema in mice, suggesting a suppression of the release of inflammatory mediators such as PGE2 and COX-2.

### 3.9. Rhododendron tomentosum *Harmaja*

*Rhododendron tomentosum* Harmaja (Ericaceae) is a small evergreen shrub that is widely distributed in northern and central Europe, North America, and northern Asia. This species has been widely used in folk medicine against arthrosis, rheumatism, pain, wounds, fever, and cough, even if the internal use of its extracts has become rare due to it containing the toxic sesquiterpenoid ledol [44]. The antiarthritic potential of this species was recently assessed by Jesionek and coworkers [45]. The authors tested the antiproliferative activity of different EOs from *R. tomentosum* on CD4+ and CD8+ limphocytes, which are involved in the pathogenesis of rheumatoid arthritis. The pro-apoptotic effects on synovia-infiltrating limphocytes and monocytes or macrophages, which impact rheumatoid-arthritis-affected joint synovium, were also evaluated. In both experiments promising results were obtained.

### 3.10. Strobilanthus ixiocephala *Benth*

Agarwal and coworkers reported the in vivo anti-inflammatory and antiarthritic activities of the EO from *Strobilanthus ixiocephala* Benth. flowering tops [46]. This small straggling shrub is present in India and belongs to the Acanthaceae family [47]. The plant species was collected in Nashik, India, and the EO was mainly constituted by the sesquiterpene alcohols ixiocephol and Τ-cadinol, the monoterpenoid alcohols isoborneol and α-fenchol, and the sesquiterpene β-caryophyllene. Beside the dose-related anti-inflammatory potential demonstrated in both acute and chronic models of inflammation, carragenan-induced rat paw edema, and cotton pellet granuloma, *S. ixiocephala* EO was effective in Freund’s adjuvant-induced arthritis in rats. At the oral dose of 1 mL/Kg, it inhibited the rat paw edema by 35.56% after 21 days of treatment. The EO was able to suppress the increased lymphocyte count in arthritic rats and the migration of leucocytes into the inflamed area [46].

### 3.11. Zingiber officinale *Roscoe*

Ginger (*Zingiber officinale* Roscoe, Zingiberaceae) is a well-known and widely utilized spice plant. This species has been traditionally used for centuries in Chinese, Ayurvedic, and Tibb-Unani medicines for the treatment of different diseases, including rheumatoid arthritis [48,49]. Initially, the studies concerning the antiarthritic effects of this plant species focused on its main phenolic secondary metabolites, gingerols. A potent antiarthritic effect has been demonstrated for gingerol-containing extracts using the streptococcal cell wall (SCW)-induced arthritis model [50]. Funk and coworkers evaluated the effects of ginger essential oils in female Lewis rats with SCW-induced arthritis [51]. The tested sample consisted of a lipophilic sesquiterpene-containing gingerol-free fraction of a dichloromethane extract of ginger rhizome. When injected at a dose 28 mg/kg/d, it was able to inhibit the chronic phase of arthritis (days 13–28), but no effects were detected on acute joint swelling.

### 3.12. Other Investigated Essential Oils

Zhang and coworkers recently evaluated the antiarthritic potential of Zingiberaceae plant EOs, including *Alpinia galanga* (L.) Willd., *Alpinia oxyphylla* Miq., *Amomum kravanh* Pierre ex Gagnep., and *Kaempferia galanga* L. [52]. These species were demonstrated to down-regulate the expression levels of COX-2, TNF-α, IL-6, and IL-1 in Freund’s adjuvant-induced arthritic rats, with *K. galanga* being the most effective one.

### 3.13. Ointment Containing a Mixture of Essential Oils

Komeh-Nkrumah and coworkers tested the antiarthritic potential of an ointment containing essential oils from 16 species: *Calophyllum inophyllum* L. (Clusiaceae), *Citrus aurantium* L. (Rutaceae), *Eucalyptus globulus* Labill. (Myrtaceae), *Eugenia caryophyllata* L. (Myrtaceae), *Foeniculum vulgare* L. (Apiaceae), *Helichrysum angustifolium* DC. (Asteraceae), *Juniperus virginiana* L. (Cupressaceae), *Lavandula angustifolia* Mill. (Lamiaceae), *Myristica fragrans* Houtt. (Myristicaceae), *Ocimum basilicum* L. (Lamiaceae), *Pinus sylvestris* L. (Pinaceae), *Piper nigrum* L. (Piperaceae), *Rosmarinus officinalis* L. (Lamiaceae), Salvia sclarea L. (Lamiaceae), *Salvia officinalis* L. (Lamiaceae), and *Zingiber officinale* Roscoe [53]. EOs were used in different percentages, and were combined with corn oil as a carrier oil, as well as with bees wax. This ointment containing 20% EO was applied topically twice daily to Lewis rats to evaluate the effects on adjuvant arthritis, which was induced by injecting *M. tuberculosis* (Mtb). It was observed that arthritic rats treated with the EO mixture developed less severe clinical arthritis compared to the control group. Moreover, the treatment was able to significantly reduce the levels of TNF-α and IL-1β, as well as the activity of matrix metalloproteinases (MMPs) in the synovial fluid (SF) and synovium-infiltrating cell (SIC) lysate.

Studies on the antiarthritic activity of EOs reported in this review are summarized in Table 1.

## 4. Essential Oils Main Components with Potential Antiarthritic Activity

Essential oils are complex mixtures containing low molecular weight components, whose extraction is above all carried out by steam distillation. The main chemical constituents in EOs include terpenoids and phenylpropanoids. Additionally, several aromatic and aliphatic compounds are also present. Monoterpenes, sesquiterpenes, and their oxygenated derivatives are the major groups of EOs chemical compounds [21].

The antiarthritic potential of terpenes, the main components in EOs, has been reviewed recently by Carvalho and colleagues [54]. An in vivo beneficial effect was reported for 24 terpenes, such as the triterpene emodinol isolated from *Paeonia emodi* Wall., and the sesquiterpene torilin, which were both demonstrated to modulate the levels of pro-inflammatory cytokines TNF-α, IL-1β, and IL-6 in vivo.

**Figure 2:**
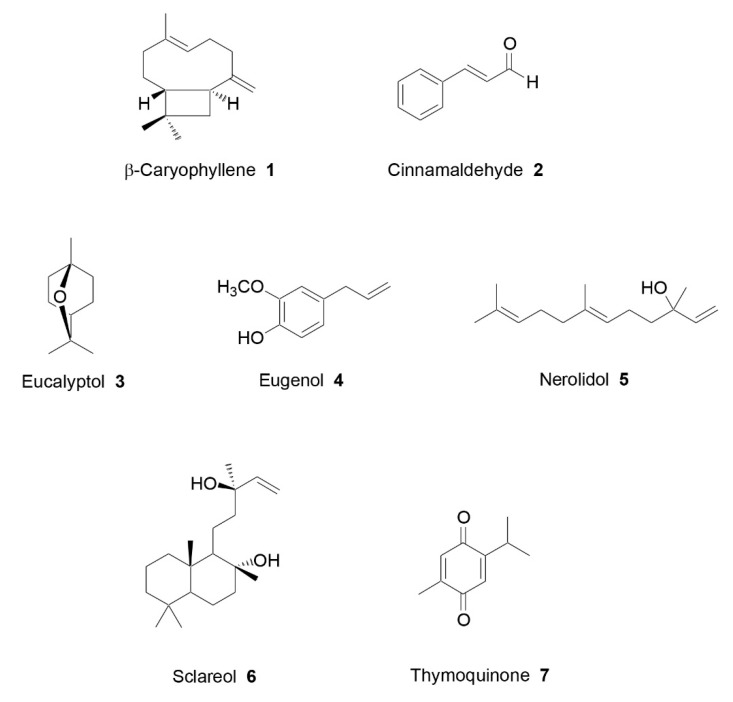
EO chemical components with antiarthritic potential [55,56,57,58,59,60,61,62,63,64].

Even more recently, other interesting terpenes, such as nerolidol, were described for their beneficial effects on arthritic conditions. The present review will focus on these further terpenes and other kinds of EOs components, which were investigated through both in vivo and in vitro assays. Seven EOs chemical constituents have been recently described for their potential antiarthritic activity (Figure 2).

### 4.1. β-Caryophyllene

β-caryophyllene (Figure 2) is a bicyclic sesquiterpene commonly found in a great number of plant species, which is present in essential oils from various spices, fruits, and ornamental plants [65]. This terpene is defined as a phytocannabinoid, which has been approved by European agencies and the Food and Drug Administration (FDA) as a flavoring agent, food additive, and taste enhancer [66].

Vijayalaxmi and colleagues verified the effectiveness of this compound on FCA-induced arthritic rats [55]. Paw volume and biochemical parameters were evaluated, and β-Caryophyllene showed antiarthritic activity. The histopathology and radiology also confirmed the anti-inflammatory activity of this molecule.

More recently, El-Sheikh and coworkers assessed the ability of β-caryophyllene to increase the efficacy of methotrexate and leflunomide and to improve their side effects [56]. Experiments were carried out on FCA-induced arthritic rats. The co-administration of β-caryophyllene and methotrexate or leflunomide significantly improved the therapeutic efficacy of these two drugs and also reduced their myelosuppressive and hepatotoxic effects, thus suggesting that β-caryophyllene could be utilized with these two drugs to reduce their side effects or increase their efficacy

### 4.2. Cinnamaldehyde

Cinnamaldehyde (*trans*-cinnamic aldehyde) is known to be the principal component of cinnamon flavor and a potent antimicrobial compound. It is also detectable in other EOs and is commonly used as a natural food flavorant [67,68].

Different studies underlined the anti-inflammatory activity of this aldehyde, which has been demonstrated to suppress the production of NO, TNF-α, and PGE2 in lipopolysaccharide (LPS)-stimulated macrophages [69].

Cheng and coworkers reported the interesting antiarthritic effects of cinnamaldehyde [57]. At concentrations of 60 and 80 nM, it was able to significantly suppress IL-1β-induced cytokine production in MH7A cells via the suppression of JAK/STAT pathways. Moreover, cinnamaldehyde was demonstrated to have beneficial effects on collagen-induced arthritis in rats.

The antiarthritic activity was also evaluated in a rat model of arthritis by Mateen and colleagues [58]. Cinnamaldehyde (10 and 20 mg/kg/day) was administered orally for 15 days in collagen-induced arthritic rats. This compound was able to reduce ROS and NO levels and to increase the reduced glutathione level in arthritic rats. It was able to improve the levels of TNF-α, IL-6, and IL-10. The effectiveness of cinnamaldehyde in decreasing the severity of arthritis was also confirmed by histopathological and radiological findings.

### 4.3. Eucalyptol

The monoterpene oxide eucalyptol (1,8-cineol), the principal component of the essential oils from Eucalyptus leaves [70], is well-known for its anti-inflammatory [71,72] and analgesic properties [73].

Yin and coworkers recently verified the anti-inflammatory and analgesic potential of this compound on a mouse model of gout arthritis [59]. The disease was induced by the injection of MSU crystals into the ankle joint. Eucalyptol was able to reduce MSU-induced allodynia, edema, and neutrophil infiltration in ankle joint tissues. Moreover, this molecule inhibited nucleotide-binding oligomerization domain (NOD)-, leucine-rich repeat (LRR)-, and pyrin domain-containing protein 3 (NLRP3) inflammasome activation and the production of pro-inflammatory cytokines. The oxidative stress induced by MSU in both RAW 264.7 cells in vitro and in ankle joint tissues in vivo was also reduced.

### 4.4. Eugenol

Eugenol is present in numerous aromatic plants, such as *Myristica fragrans* Houtt. [74] and *Ocimum basilicum* L. [75]. The clove plant (*Eugenia caryophyllata* Thunb.) is the principal source of this compound, representing 45–90% of the total EOs. A wide spectrum of biological properties has been reported for this molecule, including antioxidant, antimicrobial, anti-inflammatory, analgesic, and anticancer activities [76].

The efficacy of this allylbenzene in arthritic rats was verified by Grespan and colleagues [60]. Arthritis was induced in mice through the injection of an emulsion of bovin collagen type II and complete Freund’s adjuvant. Here, 100 µg of eugenol/day were administered orally. This compound was demonstrated to inhibit mononuclear cell infiltration and to decrease the cytokine (TNF-α, IFN-γ, and TGF-β) levels within the ankle joints.

### 4.5. Nerolidol

Nerolidol is a naturally occurring sesquiterpene alcohol used in cosmetics and as a food-flavoring agent [77,78]. It has been detected in the EOs from more than 30 plant species, such as *Ginkgo biloba* L. [79], *Piper claussenianum* (Miq.) C. DC. [80], *Momordica charantia* L. [81], and *Baccharis dracunculifolia* DC. [82]. Different biological activities have been described for this terpenoid, such as antioxidant, antifungal, antiparasitic, antinociceptive, anxiolytic, antitumor, and repellent activities [78].

Nano-encapsulated nerolidol was recently studied for its effects on zymosan-induced arthritic mice [61]. An in vivo neutrophil migration assay was performed and both free nerolidol and its polymer nanoparticles pointed out a significant anti-inflammatory activity in arthritic mice, improving the levels of anti-inflammatory cytokines and significantly inhibiting the neutrophil migration into the joint cavity.

### 4.6. Sclareol

Sclareol s a labdane diterpene ditertiary alcohol of high value in the fragrance industry, as this molecule is a good starting material for the semisynthesis of different commercial substances due to its labdane carbon skeleton and the two hydroxyl groups [83]. This terpene is a major component in the EO of clary sage (*Salvia sclarea* L.) [84], but it is also present in other plant species, such as *Cleome spinosa* Jacq. [85] and *Cistus creticus* L. [86]. Sclareol is known to possess anti-inflammatory properties—it has been demonstrated to inhibit NO production and iNOS and COX-2 expression in LPS-stimulated macrophages, and to reduce paw edema and neutrophil infiltration in λ-carrageenan-induced paw edema model [87].

Zhong and colleagues assessed the anti-osteoarthritic properties of sclareol in IL-1β-induced rabbit chondrocytes and in a rabbit model of osteoarthritis induced by ACLT [62]. Sclareol inhibited MMP, iNOS, and COX-2 expression, while increasing the TIMP-1 expression in vitro. It was also able to ameliorate cartilage degradation in vivo.

Tsai and coworkers assessed the potential therapeutic effects of sclareol on RA using the human synovial cell line SW982 and the collagen-induced arthritis (CIA) mouse model [63]. The authors demonstrated that this compound can reduce the IL-1β-induced expression of TNF-α, MMP-1, and IL-6 in the SW982 cell line via attenuating NF-κB translocation and the phosphorylation of MAPK pathways. Moreover, it was demonstrated that sclareol (5 and 10 mg/kg intraperitoneally) was able to improve swelling and bone erosions, and a reduction in the number of Th17 cells was also observed.

### 4.7. Thymoquinone

The monoterpene thymoquinone is the major constituent of *Nigella sativa* L. seeds EO [88]. Antioxidant, anti-inflammatory, antimicrobial, antiparasitic, anticancer, and hypoglycemic activities have been highlighted for this compound [89].

Tekeoglu and coworkers investigated the anti-inflammatory effects of this monoterpene on arthritis in rat model [64]. Arthritis was induced by Freund’s incomplete adjuvant and thytmoquinone was administered orally (2.5 and 5 mg/kg). It was demonstrated that the blockade of pro-inflammatory cytokines, such as TNF-α and IL-1β, reduced the severity of the disease.

## 5. Mechanisms of Action

The in vitro protein denaturation method using bovine serum albumin as a protein model has often been used to assess the antiarthritic potential of some of the EOs reported here, while their anti-inflammatory potential has generally been assessed using a rat ear edema model.

The mechanism of action underlying the antiarthritic activity of some of the investigated species reported above was more deeply investigated. In some cases, the different expression levels of pro-inflammatory cytokines and COX-2 in inflamed synovial membranes among control and treated groups were verified. As a results of their investigations, Suh et al. demonstrated that *C. obtusa* was able to inhibit the expression of IL-1β, TNF-α, and IL-6 in the inflamed synovial membrane, as well as IL-1β and IL-6 in an inflamed meniscus [32]. Such effects were also demonstrated for *Litsea cubeba*, whose EO was able to decrease the TNF-α, IL-1β, IL-6, IL-8, and IL-17A levels and to increase IL-10 in type II collagen-induced arthritic rats [40]. *K. galanga* and some other species belonging to the Zingiberaceae family also demonstrated impacts on the expression of COX-2, TNF-α, IL-6, and IL-1 in Freund’s adjuvant-induced arthritic rats [52]. The levels of TNF-α and IL-1β and the activity of MMPs in the synovial fluid were also reduced by an ointment containing essential oils from 16 species, among which were *E. caryophyllata*, *C. inophyllum*, *C. aurantium*, and *E. globulus*, as described by Komeh-Nkrumah and coworkers [53].

Furthermore, Yamada and coworkers [43] demonstrated that oral treatment with *O. americanum* EO (150 mg/kg) for seven days in arthritic mice was able to inhibit leukocyte migration in the synovial membrane and to attenuate cartilage destruction. Moreover, the treatment significantly reduced IFN-γ levels in synovial tissue.

*R. tomentosum* EO was demonstrated to have antiproliferative and pro-apoptotic activity toward CD4 and CD8 T cells. The same effects were also observed toward synovia-infiltrating monocytes and macrophages [45].

The mechanisms of action of some EOs’ pure components were also investigated. Cinnamaldehyde, for example, has been demonstrated to significantly suppress IL-1β-induced inflammatory cytokine production in human synoviocyte cell line MH7A via the suppression of JAK/STAT pathways [57] and to improve the TNF-α, IL-6, and IL-10 levels in arthritic rats [58]. Eugenol was also demonstrated to decrease the levels of TNF-α, IFN-γ, and TGF-β in induced arthritic rats [60]. Lastly, Tsai and coworkers demonstrated that sclareol can reduce the expression of MMP-1, TNF-α, and IL-6 in SW982 cells via the phosphorylation of MAPK pathways [63].

## 6. Essential Oils and Their Chemical Components in the Treatment of Arthritis-Related Pain

EOs and their chemical components could also be useful in the treatment of arthritis-related pain. Various monoterpenes, sesquiterpenes, and other essential oil constituents have been investigated for their potential antinociceptive activity and have demonstrated interesting analgesic-like activity [90] and different species demonstrated antinociceptive activity in animal models of nociception [91]. For example, *M. spicata* L. EO and its main constituents, such as menthol, carvone, and limonene, demonstrated analgesic effects in animal models, while spearmint oil was able to reduce pain in osteoarthritis patients [92]. Recently, Mota and colleagues reported the analgesic effect of citral [93], a monoterpene aldehyde identified in the EOs of several plants, such as lemongrass (*Cymbopogon citratus* (DC.) Stapf.) [94] and ginger (*Zingiber officinale* Roscoe) [95,96]. It was demonstrated that a single administration of citral (at concentrations of 100 and 300 mg/kg) reversed FCA-induced mechanical allodynia in arthritic rats [93].

## 7. Negative Results

*Curcuma longa* L. (Zingiberaceae) is known to contain two main classes of secondary metabolites, phenolic curcuminoids, and essential oils, including the major components α-turmerone and β-turmerone. Despite the essential oil from *Curcuma longa* L. administered via intraperitoneal injection demonstrating a strong antiarthritic effect in female rats with SCW-induced arthritis, significant morbidity and mortality have been observed, which do not support the use of this EO against arthritis [97]. On the contrary, a good antiarthritic effect has been demonstrated in experimental rheumatoid arthritis for curcuminoid-containing turmeric extracts [98], and good antiarthritic potential has been observed for curcumin, the principal curcuminoid of turmeric. Nonose and coworkers demonstrated that this compound was able to reduce the inflammatory response in zymosan-induced arthritis [99].

## 8. Concluding Remarks

The low toxicity and reduced genotoxicity are some of the advantages associated with the use of EOs [21]. However, due to the structural relationships within each chemical group, the constituents of EOs may undergo cyclization, isomerization, oxidation, and dehydrogenation reactions, which can easily convert them into each other. As a consequence, the chemical compositions of EOs are influenced not only by factors affecting the plant material, such as the growth stage and habitat, but also by the conditions during both the processing and storage of the plant, during distillation, and during the subsequent handling of thee EOs. Their chemical components are subjected to oxidative damage, chemical transformations, and polymerization reactions that generally lead to sensory and pharmacological properties loss. These deterioration reactions have not been completely addressed and need to be taken into account [100].

Moreover, some old essential oils and oxidized terpenoids have demonstrated skin-sensitizing activity causing contact dermatitis. A study of the toxicity profile of EOs should be carried out, even if this kind of investigation is not simple due to the variability of EO compositions, which in turn may be affected by a number of factors [21,100].

The application of EOs and their constituents in the treatment of arthritis appears to be an interesting new perspective on their potential health benefits. A total of 31 plant EOs and some chemical compounds commonly distributed in different EOs have been reported in the literature to have in vitro or in vivo antiarthritic potential. Many other EOs and components could still be potentially identified.

Overall, the analysis of the existent scientific literature focusing on the antiarthritic potential of EOs reveals that the information obtained by in vitro and in vivo studies still needs to be confirmed through clinical investigations.

## Figures and Tables

**Table 1:** Essential oils investigated for their antiarthritic potential.

Plant Species	Common Name	Family	Part	Study	Animal Model/In Vitro Test	Ref.
*Alpinia galanga* (L.) Willd.	greater galangal	Zingiberaceae	n.s. ^1^	In vivo	Freund’s adjuvant-induced arthritic rats	[52]
*Alpinia oxyphylla* Miq.	black cardamom	Zingiberaceae	n.s.	In vivo	Freund’s adjuvant-induced arthritic rats	[52]
*Amomum kravanh* Pierre ex Gagnep.	cardamom	Zingiberaceae	n.s.	In vivo	Freund’s adjuvant-induced arthritic rats	[52]
*Aquilaria agallocha* Roxb.	agarwood	Thymelaeaceae	Heartwood	In vitro	BSA denaturation method	[28]
				In vivo	Freund’s adjuvant-induced arthritic rats	
*Calophyllum inophyllum* L.	Alexandrian laurel	Clusiaceae	n.s.	In vivo	Mixture of essential oils tested on adjuvant arthritis in Lewis rats	[53]
*Cedrus deodara* (Roxb.) Loud.	deodar	Pinaceae	Wood	In vivo	Freund’s adjuvant-induced arthritis in rats	[31]
*Chamaecyparis obtusa* (Siebold and Zucc.) Endl.	hinoki cypress	Cupressaceae	Leaves	In vivo	Carrageenan-induced arthritis in rats	[32]
*Citrus aurantium* L.	bitter orange	Rutaceae	n.s.	In vivo	Mixture of essential oils tested on adjuvant arthritis in Lewis rats	[53]
*Cyperus esculenthus* L.	yellow nutsedge	Cyperaceae	n.s.	In vivo	Formaldehyde-induced arthritis in rats	[33]
*Cyperus rotondus* L.	yellow nutsedge	Cyperaceae	n.s.	In vivo	Formaldehyde-induced arthritis in rats	[33]
*Eucalyptus globulus* Labill.	Tasmanian blue gum	Myrtaceae	n.s.	In vivo	Mixture of essential oils tested on adjuvant arthritis in Lewis rats	[53]
*Eugenia caryophyllata* L.	clove	Myrtaceae	n.s.	In vivo	Mixture of essential oils tested on adjuvant arthritis in Lewis rats	[53]
*Foeniculum vulgare* L.	sweet fennel	Apiaceae	n.s.	In vivo	Mixture of essential oils tested on adjuvant arthritis in Lewis rats	[53]
*Gaultheria fragrantissima* Wall.	wintergreen	Ericaceae	Leaves	In vitro	Protein denaturation method; egg albumin denaturation method	[35]
*Helichrysum angustifolium* DC.	everlasting	Asteraceae	n.s.	In vivo	Mixture of essential oils tested on adjuvant arthritis in Lewis rats	[53]
*Juniperus virginiana* L.	red cedar	Cupressaceae	n.s.	In vivo	Mixture of essential oils tested on adjuvant arthritis in Lewis rats	[53]
*Kaempferia galanga* L.	kencur	Zingiberaceae	n.s.	In vivo	Freund’s adjuvant-induced arthritic rats	[52]
*Lagerstroemia speciosa* (L.) Pers.	banaba	Lythraceae	Leaves	In vitro	Protein denaturation method	[38]
*Lavandula angustifolia* Mill.	lavender	Lamiaceae	n.s.	In vivo	Mixture of essential oils tested on adjuvant arthritis in Lewis rats	[53]
*Litsea cubeba* (Lour.) Pers.	mountain pepper	Lauraceae	Fruits	In vivo	Collagen-induced arthritic rats	[40]
*Myristica fragrans* Houtt.	nutmeg	Myristicaceae	n.s.	In vivo	Mixture of essential oils tested on adjuvant arthritis in Lewis rats	[53]
*Ocimum americanum* L.	American basil	Lamiaceae	Leaves	In vivo	Zymosan-induced arthritis in mice	[43]
*Ocimum basilicum* L.	basil	Lamiaceae	n.s.	In vivo	Mixture of essential oils tested on adjuvant arthritis in Lewis rats	[53]
*Pinus sylvestris* L.	Scots pine	Pinaceae	n.s.	In vivo	Mixture of essential oils tested on adjuvant arthritis in Lewis rats	[53]
*Piper nigrum* L.	black pepper	Piperaceae	n.s.	In vivo	Mixture of essential oils tested on adjuvant arthritis in Lewis rats	[53]
*Rhododendron tomentosum* Harmaja	marsh Labrador tea	Ericaceae	shoots	In vitro	Peripheral blood lymphocytes of healthy volunteers; synoviocytes and immune cells isolated from synovia of RA patients	[45]
*Rosmarinus officinalis* L.	rosemary	Lamiaceae	n.s.	In vivo	Mixture of essential oils tested on adjuvant arthritis in Lewis rats	[53]
*Salvia sclarea* L.	clary sage	Lamiaceae	n.s.	In vivo	Mixture of essential oils tested on adjuvant arthritis in Lewis rats	[53]
*Salvia officinalis* L.	sage	Lamiaceae	n.s.	In vivo	Mixture of essential oils tested on adjuvant arthritis in Lewis rats	[53]
*Strobilanthus ixiocephala* Benth.	waiti	Acanthaceae	Flowering tops	In vivo	Freund’s adjuvant-induced arthritis in rats	[46]
*Zingiber officinale* Roscoe	ginger	Zingiberaceae	Rhizome	In vivo	SCW-induced arthritis	[51]
			n.s.	In vivo	Mixture of essential oils tested on adjuvant arthritis in Lewis rats	[53]

Note: ^1^ n.s. not specified.

## Abbreviations

ACLT	anterior cruciate ligament transection
CIA	collagen-induced arthritis
COX-2	cyclooxygenase-2
DMARDs	disease-modifying antirheumatic drugs
EO	essential oil
FCA	Freund’s complete adjuvant
FDA	Food and Drug Administration
IFN-γ	interferon-γ
IL-1β	interleukin-1β
IL-6	interleukin-6
iNOS	inducible nitric oxide synthase
JAK/STAT	Janus kinase/signal transducer and activator of transcription
LPS	lipopolysaccharide
MMPs	matrix metalloproteinases
MSU	monosodium urate
Mtb	*M. tuberculosis*
NLRP3	NOD-, LRR-, and pyrin-domain-containing 3
NO	nitric oxide
NSAIDs	non-steroidal anti-inflammatory drugs
OA	osteoarthritis
PGE2	prostaglandin E2
RA	rheumatoid arthritis
SCW	streptococcal cell wall
SF	synovial fluid
SIC	synovium-infiltrating cells
SW982	human synovial cell line
TGF	tumor growth factor
TIMPs	tissue inhibitors of metalloproteinases
TNF-α	tumor necrosis factor
